# The Impact of Dance-Based Physical Activity on Sensorimotor and Psychological Function in Parkinson’s Disease: A Narrative Review

**DOI:** 10.3390/healthcare14010105

**Published:** 2026-01-01

**Authors:** Giuditta Carretti, Lorenzo Guidi, Mirko Manetti, Mirca Marini

**Affiliations:** Section of Anatomy and Histology, Department of Experimental and Clinical Medicine, University of Florence, 50134 Florence, Italy; giuditta.carretti@unifi.it (G.C.); lorenzo.guidi5@edu.unifi.it (L.G.); mirko.manetti@unifi.it (M.M.)

**Keywords:** neurodegenerative disorder, integrative medicine, dance therapy, rhythmic movement, psychophysical reeducation, adapted kinesiology, functional mobility, self-efficacy, quality of life

## Abstract

**Background and Objectives**: Parkinson’s disease (PD) is a progressive neurodegenerative disorder characterized by a wide range of motor and non-motor symptoms that significantly compromise daily functionality, psychophysical wellbeing, and quality of life. Currently, a number of pharmacological and surgical treatments can reduce the clinical severity of motor impairments, but they are limited or poorly tolerated for non-motor symptoms, thus highlighting the need for non-medical complementary approaches. In this context, dance-based interventions have emerged as promising and enjoyable integrative strategies to globally and safely manage such multidimensional complex challenges. This narrative review aims to synthesize the current evidence of the effectiveness of dance-based interventions to improve psychophysical function and quality of life in individuals affected by PD, also providing an updated insight into the feature-related benefits of different dance styles. **Methods**: A comprehensive literature search of PubMed, Scopus, Web of Science, and Cochrane was conducted, and 66 original studies investigating dance-based integrative interventions to enhance physical, cognitive, and socioemotional outcomes in this target population were selected. **Results**: Across different styles, the reviewed literature suggests that dance can positively impact on motor symptoms, neuroplasticity, and psychosocial outcomes through rhythmic cueing, motor–cognitive integration, and expressive and social engagement. Furthermore, dance offers a non-medicalized enjoyable context able to foster motivation and practice adherence. **Conclusions**: Dance-based interventions represent a promising complementary approach in the management of PD, with the potential to enhance both physical functioning and overall quality of life. Further rigorous, longitudinal and comparative studies are needed to clarify dose–response relationship, style-specific effects, and long-term benefits.

## 1. Introduction

Neurological diseases represent the second leading cause of morbidity and mortality worldwide and, among them, Parkinson’s disease (PD) is the second most prevalent neurodegenerative disorder in adult and elderly population [1,2]. According to recent global estimates, PD affects approximately six million individuals and such rate is expected to double in the next 2–3 decades [3,4,5]. It is an age-related neurological disorder characterized by the progressive loss of dopaminergic neurons in the substantia nigra pars compacta that leads to dopamine deficiency in the basal ganglia circuitry [6,7]. The neurodegenerative process clinically manifests through a wide spectrum of motor symptoms such as bradykinesia, rigidity, resting tremor, gait alterations, and postural instability. Non-motor symptoms including cognitive decline, depression, anxiety, apathy, sleep disorders, and autonomic dysfunctions are documented as well [2,8,9]. Given the complex multidomain nature of these impairments, PD progressively jeopardizes psychophysical functioning drastically reducing self-efficacy, autonomy, overall wellbeing, and quality of life [10,11,12]. As the disease progresses, daily life tasks and recreational activities become increasingly challenging hence fueling family dependence, frustration, social isolation, and sedentariness [13]. 

Bradykinesia, consisting in slowness of movement initiation and execution accompanied by impaired motor control and decreased movement amplitude, is considered the core diagnostic criterion of PD [14,15]. In particular, repetitive and alternating movements are the most impacted with progressively diminished automaticity leading to functional disability and jeopardizing self-efficacy in daily life activities [16]. Regarding sensorimotor control, a reduced responsiveness to external cues is often experienced by this target population thus negatively influencing their interaction with the surrounding environment and other individuals [17,18]. Rigidity typically manifests as uniform or oscillatory increased resistance to passive movement attended by a tremor component, which results in the typical PD impairment known as cogwheel rigidity [15,19]. Consequently, there is a reduction in trunk mobility and upper body posture alignment/control, as well as a decrease in arm swing [14,20]. Resting tremor, the most noticeable motor sign that mainly affects distal extremities, usually presents an asymmetric onset and loses intensity during voluntary movements [21]. In the advanced stages of disease, deficits in postural reflexes and adjustments to perturbations generally emerge drastically compromising gait stability and efficiency. Clinically, such disorders manifest as shuffling/festinating steps with frequent episodes of freezing of gait that hinder the subject from initiating or continuing walking despite the intention to move [22,23,24]. Many individuals also experience an impaired motor coordination of facial expression, speech, and handwriting thus resulting in hypomimia, hypophonia, and micrographia, respectively [14]. 

All these complex and multidimensional motor symptoms significantly increase fall risk, functional dependence, and sedentariness with a negative impact on the overall quality of life and healthcare costs [25,26,27]. Therefore, early diagnosis, multidomain comprehensive assessment, and tailored treatment strategies become crucial in this field [7,28]. Currently, pharmacological (e.g., levodopa and dopamine agonists) and neurosurgical treatments represent the mainstay in managing motor symptomatology but provide limited or inconsistent relief for neuropsychiatric impairments [10,29]. Moreover, long-term pharmacotherapy is often associated with adverse effects that are able to further undermine the overall health-related quality of life [30]. Such limitations and the urgent need to globally reduce sociomedical charges have progressively posed interest in investigating and applying integrative therapeutic approaches within PD care [31,32,33]. Therefore, non-medical interventions targeted to holistically improve motor, cognitive, and social domains, enhance psychophysical functioning, and foster long-term adherence to active lifestyles have increasingly gained emphasis [2,33,34,35,36]. 

In this context, adapted physical activity has emerged as a promising tool to address and counteract the wide spectrum of PD-related sequelae and challenges [4,29,37,38,39,40]. In fact, there is accumulating evidence proving the beneficial role of structured exercise in ameliorating functional capacity and psychosocial wellbeing while attenuating cognitive decline and mood fluctuations in this target population [41,42,43]. By fostering neuroplasticity and modulating neurotransmitter systems, as well as neurotrophic factors implicated in PD pathophysiology, physical activity becomes a crucial component within the aforementioned integrative approach [44,45,46]. Despite this growing evidence, most people do not meet the recommended exercise guidelines due to real or perceived barriers regarding fear of falling, fatigue, lack of specialized professionals providing enjoyable proposals, and scarce peer/family motivation and support [47,48]. In detail, exercise guidelines for PD recommends 150 min per week of moderate to vigorous intensity exercise including aerobic and resistance training integrated by balance and mobility exercises [49,50].

In particular, activities combining physical, sensory, and cognitive tasks, such as dance, seem to produce amplified synergistic effects on all the complex domains deteriorated by the onset and progression of disease [51,52,53]. Dance combines aerobic exercise, coordinative patterns, rhythmic cues, cognitive involvement, and socio-emotional expression/connection, thus counteracting the complex PD-related deficits through multidimensional stimuli [53,54]. Regular practice promotes sensorimotor integration, gait reeducation and balance control, as well as psychological wellbeing, within a supportive non-medicalized context [32,33,55,56]. Furthermore, compared with conventional exercise, dance shows higher effectiveness in fostering long-term adherence to practice [57]. Such features, mostly linked to the enjoyable, social, and holistic engaging nature of dance, become crucial in chronic neurodegenerative disorders [31,57,58]. Finally, owing to the wide range of available styles, dance-based interventions can safely be tailored to the variegated needs of this vulnerable population and the different stages of disease [59,60].

On this basis, the present narrative review aims to summarize and critically analyze current literature on dance-based interventions in PD. Specifically, this work focused on their impact on sensorimotor and psychosocial outcomes while contemporarily seeking to provide insight into the potential benefits of different dance styles. Hopefully, our evidence overview might further foster on-field application of dance as a safe, engaging, and promising integrative tool in the multidisciplinary complex management of PD.

## 2. Search Strategy and Evidence Selection

**Figure 1:**
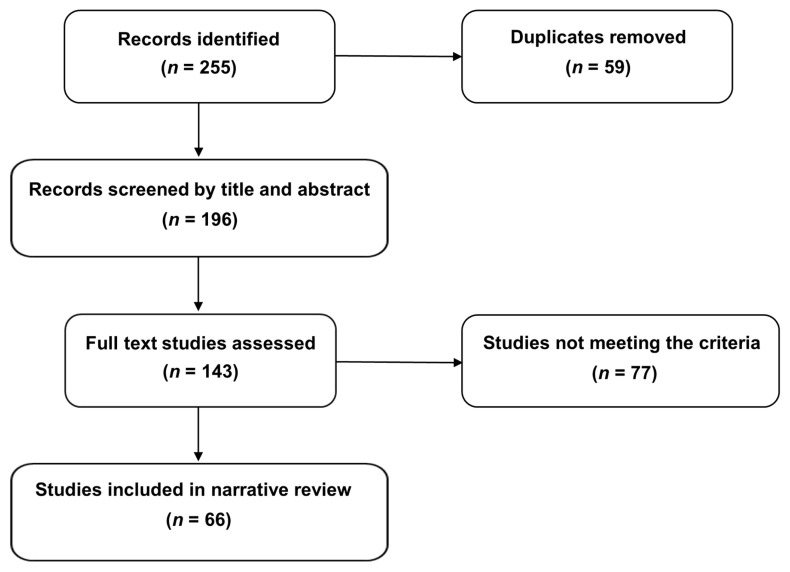
Flow diagram of literature search and study selection.

A comprehensive search of MEDLINE via PubMed and Scopus, Web of Science, and Cochrane databases was performed to detect eligible studies focusing on dance-based interventions addressing PD-affected individuals. The following keywords and medical subject headings (MeSH) terms were used: “dance-based intervention in Parkinson’s disease”, “dance-based movement in Parkinson’s disease”, “dance movement therapy in Parkinson’s disease”, “therapeutic dance for Parkinson’s disease”, “rhythmic movement for Parkinson’s disease”, “exercise dance for Parkinson’s disease”, “dance style* for Parkinson’s disease”, “group dance in Parkinson’s disease”, “tango AND Argentine tango in Parkinson’s disease”, “contemporary dance for Parkinson’s disease”, “ballet in Parkinson’s disease”, “improvisational dance in Parkinson’s disease”, and “ballroom dance in Parkinson’s disease”. “Similar articles” provided by the searched databases were also screened. Finally, the same searching terms were also entered into Litmaps to identify any further research related to the investigated topic. All authors cooperated to create and refine the search strategy, and all screened the retrieved list of articles to avoid missing any relevant evidence. The search was restricted to peer-reviewed English language papers published from 2005 to 31 October 2025. Given the descriptive nature of this review and its main goals, no restrictions related to the applied dance style, frequency, duration, or setting of the intervention were applied. Original studies investigating sensorimotor and psychological outcomes relevant to PD management were analyzed in depth. The included literature predominantly comprised interventional studies, including randomized controlled trials, non-randomized controlled trials, and single-group pre–post intervention studies. A smaller number of quasi-experimental and observational cohort studies were also considered when they provided relevant outcome data related to dance-based interventions in PD. Studies omitting any description of the administered dance exercise regimen, applying mixed (e.g., music therapy or performing arts combined with dance) and online/digital approaches, or addressing different neurodegenerative disorders beyond PD were excluded. Published abstracts without full text or conference abstracts were excluded as well. At the end of the screening process, 66 original studies were selected, full text analyzed, and their main results described in the present review (Figure 1).

## 3. Results

Overall, the reviewed literature highlighted that dance-based interventions addressing people affected by PD can provide beneficial effects both on motor and non-motor symptomatology. Specifically, significant improvements in movement synchronization, gait efficiency, postural control, cognitive tasking, socioemotional expression/interaction, and overall quality of life have been documented [51,60,61,62,63]. Several studies attributed such benefits to the peculiar multidimensional engaging environment offered by this discipline and its variegated styles [53,64,65,66]. Despite these promising findings, current literature shows relevant gaps mostly due to small sample sizes, methodological heterogeneity (widely variable study design, protocol length, intensity, and outcome assessment), lack of focus on style-specific effects, and the predominance of a few research groups regularly working and owing expertise in this complex field [67,68,69]. In detail, considerable variability was detected in duration (single-session to 24 months), frequency (1 to 3 or more sessions/week), and session length (45–90 min) [64,70,71]. Intensity was often reported qualitatively rather than with standardized physiological measures, limiting dose–response comparisons. Settings ranged from clinical and research environments to community studios, with non-medicalized contexts typically enhancing enjoyment and adherence [59,72]. Standardized reporting of these parameters is critical to interpret outcomes and optimize future protocols. Therefore, further rigorous long-term studies, also exploring dose–response relationships, as well as comparison of different dance styles, are needed.

### 3.1. Psychophysical Benefits of Dance-Based Interventions in Parkinson’s Disease

Across the analyzed studies, dance-based interventions consistently yielded significant psychophysical benefits for individuals living with PD. The main emerged core evidence regards the dance peculiarity of naturally fostering the integration of sensory, perceptual, and motor processes [73,74,75,76]. Featured by a sociorelational dimension and an expressive non performative purpose, dance also provides a more engaging environment able to mitigate exercise-related fatigue or boredom than conventional physical activity [57,77,78]. Such uniquely integrative approach offers a concrete and safe opportunity to counteract the complex multidimensional sequelae of PD while proactively involving the subject in the continuum of care. Multiple studies applying different dance styles such as Argentine tango [70,79,80,81,82,83,84], ballroom dance [85,86,87], classical ballet [88,89,90], contemporary dance [91,92,93], folk dance [94,95,96,97], urban dance [98], and community dance programs [99,100,101] highlighted improvements in the overall sensorimotor efficiency [61,67,81,102]. Despite of the style, dance requires reactive and anticipatory postural adjustments, direction changes, highly coordinated movements, whole and district body control, reactivity to multisensory input, and spatio-temporal orientation [103,104,105,106,107]. These inherent characteristics, along with structured rhythmic entrainment, may help to bypass disease-impaired basal ganglia pathways by promoting the recruitment of alternative motor circuits, internal timing, and sensorimotor integration [71,108,109]. Moreover, rhythmic timed cues provided while dancing seem to effectively foster static and dynamic balance, stride regulation, weight shifting, turning control, and spatial orientation during locomotor transitions, thus counteracting freezing/festinating episodes and fall risk [110,111,112,113,114,115]. Literature has also reported enhanced responsiveness to multisensory cues that leads to a better efficiency and consistency in their processing and the consequent motor response [64,103]. Functionally, these improved competences translate into faster motor learning, smoother movements and safer, as well as more efficient, management of complex sensorimotor tasks [51,66]. Many dance-based interventions addressing PD-affected individuals detected a relevant reduction in fear of falling along with an improvement in body schema awareness and self-efficacy, thus suggesting a pivotal role of dance in promoting movement enjoyment and active adherence to practice [66,67,92,116]. Notably, movement confidence was primarily assessed through subjective self-report measures (e.g., confidence scales and perceived balance confidence questionnaires) [27,117,118], although some studies also linked these perceptions to objective functional outcomes such as improved gait parameters, balance tests, and reduced freezing episodes [51,72,111,112]. Such distinction highlights the complementary role of perceived and performance-based measures in interpreting functional relevance [73,115,119].

Given the cognitive demands of learning choreographic sequences through imagery and imitation, psychophysically interacting with partner/peers, and following rhythm, dance positively challenges executive function, working memory, and focus hence mitigating cognitive decline [57,62,120]. It has been demonstrated that different dance styles can positively train a wide variety of cognitive skills that are crucial for daily life self-efficacy, autonomy, and social interaction [108,109,121]. In addition to the aforementioned positive effects, dance programs typically provide a social enjoyable environment able to counteract isolation and produce meaningful psychoemotional and sociorelational benefits [77,122,123,124,125]. Studies investigating dance-based intervention benefits on different psychosocial constructs in this target population reported a significant reduction in depressive symptoms and anxiety [63,107] with an improvement in the overall psychological wellbeing [53,98,118,126,127,128]. Though still heterogeneous and often lacking scientific rigor, qualitative research repeatedly highlighted that this target of individuals perceive dance classes as an enjoyable context to freely and safely experience creativity, bodily expression, and social connection [129]. Therefore, dance-based programs become a concrete opportunity to regain self-awareness, restore social identity after PD diagnosis, and defeat the disease related stigma [88,130]. Furthermore, the inherently communal structure of dance classes effectively boosts and reinforces social bonds, mutual support, sense of belonging, and emotional sharing consequently enhancing resilience and quality of life [101,131,132,133]. Both from a historical and nowadays perspective, dance has being considered a healing art showing curative properties generated by rhythm-movement combination, self-expression, and emotional connectedness [2,53,78,134]. Such beneficial effects have been primarily linked to the modulation of dopaminergic and limbic pathways stimulated by the music-driven movement and its emotionally evocative nature [67,103,135]. Longitudinal studies evidenced that these socioemotional benefits may not only boost long-term adherence but also counteract the wide spectrum of PD non-motor symptoms that hardly respond to dopaminergic therapy [33,64,69,101]. Briefly summarizing, the reviewed literature reported both acute and long-term effects of dance-based interventions for PD. In particular, acute benefits, often observed after single sessions, included transient improvements in mood, emotional regulation, perceived vitality, and responsiveness to rhythmic cues, likely driven by short-term neurochemical changes, heightened arousal, and affective engagement [56,59,98,113]. In contrast, long-term effects emerging after weeks or months of practice included more stable gains in gait, balance, postural control, cognitive performance, self-efficacy, and quality of life, presumably reflecting motor learning, neuroplastic adaptation, and behavioral habit formation [72,114,131]. Explicit differentiation among these temporal dimensions is essential to accurately interpret outcomes and optimize intervention design.

**Figure 2:**
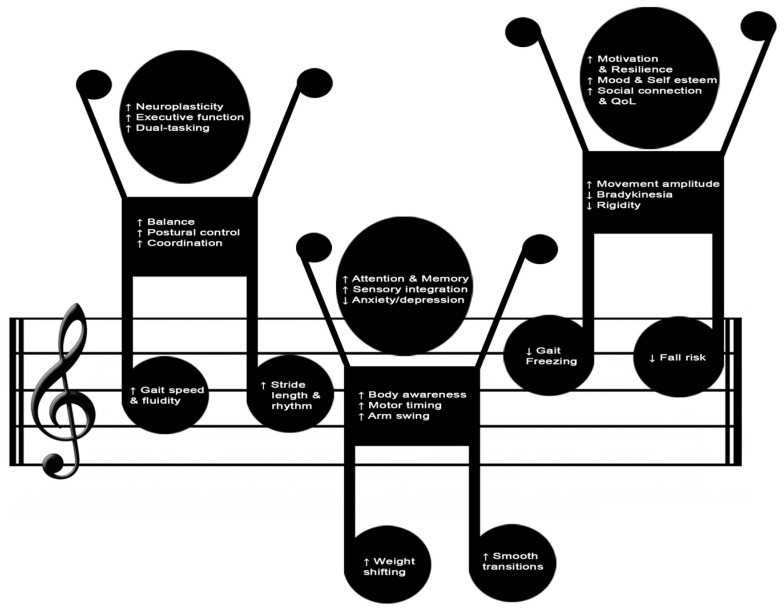
Main cognitive, psychological, and sensorimotor benefits of dance-based interventions in Parkinson’s disease.

Considering the multidimensional features of dance benefits and their crucial role in counteracting PD symptomatology, the main psychophysical positive effects are graphically summarized in Figure 2.

### 3.2. Main Applied Dance Styles and Methodologies

#### 3.2.1. Common Cross-Style Therapeutic Mechanisms

The original studies included in this narrative review investigated a wide spectrum of dance styles and methodological approaches/frameworks addressing PD-affected individuals. Each investigation presented different re-educational potentialities linked to style-specific movement features and cognitive and sociorelational tasks, as well as pedagogical principles [70,72,88,99,100,136]. Though often addressed as a research limitation, such variability actually reflects the multidimensional complexity of dance as a psychophysical, cognitive, and socioemotional activity [51,67]. Simultaneously, it also highlights the broad opportunities, offered by this discipline, to address and counteract both motor and non-motor symptomatology [61,65,137]. Dance-based interventions for PD share a set of core therapeutic mechanisms that underpin their effects across styles, including rhythmic auditory cueing, externally guided whole-body movement, balance and postural challenges, cognitive engagement, and social interaction [35,51,99]. These cross-style multimodal features address both motor and non-motor symptoms such as postural instability, gait impairment, bradykinesia, mood disturbances, and reduced quality of life [39,42,130]. Rhythmic entrainment represents a central mechanism common to most dance styles, able to facilitate gait initiation, stride regulation, and turning by compensating for impaired internal timing and movement automaticity associated with basal ganglia dysfunction [22,23,138]. Additionally, the multidirectional stepping, weight shifting, and balance demands inherent to dance directly counteract core motor deficits linked to falls and freezing of gait [20,24]. While cognitive engagement is universal, the degree of motor–cognitive coupling varies by style, reflecting differences in sequencing, attention, and executive demands [17,29]. Beyond motor effects, emotional engagement and psychosocial enrichment emerged as crucial mediators of adherence and long-term participation. Indeed, dance-based interventions consistently demonstrated higher enjoyment, motivation, and social connectedness than conventional exercise, thus fostering sustained engagement and psychological wellbeing in PD [47,48,57,60]. Owing to these mechanisms, qualitative evidence further highlighted improvements in self-efficacy, social identity, and embodiment, hence suggesting that dance uniquely addresses psychosocial dimensions of PD that are less responsive to pharmacological treatment [13,66,100,116,127]. Overall, the multisensory, socially engaging, and non-pharmacological nature of dance-based interventions may significantly improve psychophysical health and quality of life in PD [11,31,128]. In order to highlight any possible correlation between each style-specific features and the demonstrated multidimensional benefits for PD, the main administered dance styles and methodologies are detailed as follows.

#### 3.2.2. Argentine Tango

Argentine tango, characterized by a strong emphasis on partner interaction, improvisation, and multidirectional walking patterns, is one of the most extensively studied dance-based interventions in PD [51,54,70,81,82,84,87,131,139,140,141]. Unlike choreographed group dances, tango requires continuous real-time adaptation to a partner, thus imposing high demands on anticipatory postural control, reactive balance, and sensorimotor integration [72,84,105,106,139]. From a biomechanical perspective, tango uniquely incorporates backward walking, frequent starts and stops, changes in direction, and variable step lengths [23,70,72,106,107,114]. These features directly challenge gait automaticity and axial mobility, commonly impaired in PD, and may explain why tango-based interventions have been repeatedly associated with improvements in gait velocity, stride length, turning performance, and freezing of gait severity [81,84,87,112,139,142,143]. The need to respond to subtle partner cues further increases balance demands under dynamic conditions [72,80,105]. Cognitively, Argentine tango places substantial demands on executive function, attention, and decision-making. Furthermore, the improvisational component requires participants to select and modify movement strategies in real time, supporting motor–cognitive coupling and dual-task skills [17,29,64,70,79,80,144,145]. Overall, such style-specific features distinguish tango from more repetitive or instructor-led dance formats and may underlie reported improvements in cognitive flexibility, task switching, and dual-task performance [17,51,74,119,121,141,146]. Psychosocially, the close interpersonal interaction inherent to tango seems to enhance social connectedness, trust, and emotional engagement [66,82,87,99,116,131,132,147]. Moreover, the structured partner roles can foster movement confidence and self-efficacy, particularly when challenging movements are successfully negotiated within a supportive dyadic context [79,83,136]. Such relational features represent critical contributors to the relatively high adherence rates frequently reported in tango-based programs [59,60,82,133]. Overall, Argentine tango appears particularly well suited for individuals affected by PD who experience gait initiation difficulties, turning impairments, and freezing episodes, especially at mild-to-moderate disease stages [70,72,80,81,139,143]. The combination of complex motor demands, cognitive engagement, and psychosocial support uniquely positions tango as a multifaceted and promising therapeutic intervention in this target population [51,64,105,141,145]. 

#### 3.2.3. Ballroom Dance

Ballroom dance encompasses a range of partnered styles characterized by structured movement patterns, upright posture, and synchronized coordination with a partner [60,87,99,101,136]. Unlike Argentine tango, ballroom dance typically follows predefined step sequences, placing greater emphasis on rhythmic consistency, bilateral coordination, and sustained postural alignment [85,104,105,148]. Biomechanically, ballroom dance requires controlled weight shifting, symmetrical limb use, and continuous trunk engagement, which may support postural stability, balance symmetry, and endurance during prolonged movement sequences [87,101,106,119,149]. The predictable rhythmic structure can facilitate gait regularity and temporal coordination, making ballroom dance suitable for individuals who benefit from structured external pacing [85,87,99,104]. Cognitively, ballroom dance involves memorization of step patterns and coordination with musical phrasing, engaging attention and working memory without the high improvisational demands typical of tango [29,87,100,104]. This may be advantageous for participants with cognitive impairment who prefer repetitive, rule-based motor learning environments [29,86,100,115]. From a psychosocial perspective, ballroom dance promotes social bonding through partner interaction while maintaining clearer interpersonal boundaries than close-embrace tango [60,85,136,148]. Furthermore, its cultural familiarity may enhance comfort and confidence, particularly among older adults with prior dance exposure [60,87,99,150]. Overall, ballroom dance appears well suited for individuals with PD who benefit from structured, rhythmically predictable movement and who seek balance and postural improvements within a socially engaging but less cognitively demanding framework [101,105,136,148,149]. 

#### 3.2.4. Classical Ballet and Ballet Techniques

Classical ballet-based interventions are featured by their strong emphasis on posture, movement precision, and controlled execution [88,90,117,151]. Unlike partnered dance styles, ballet typically focuses on individual performance, with movements executed in relation to spatial reference points rather than interpersonal cues [104,151,152]. Biomechanically, ballet emphasizes axial alignment, core stability, controlled weight transfer, and extended joint ranges of motion, hence targeting postural control, trunk rigidity, and reduced movement amplitude due to PD [88,89,90,101]. In addition, the slow and deliberate execution of movements encourages conscious motor control and postural awareness [88,89,117]. Cognitively, ballet requires sustained attention to body position, movement accuracy, and sequencing consequently reinforcing proprioceptive awareness and motor planning [29,90,115,151,152]. This internal focus differentiates ballet from rhythm- or partner-driven styles [88,104]. Psychosocially, ballet-based programs may foster self-discipline and body consciousness, though technical demands highlight the need for appropriate adaptation and solid instructor expertise [89,90,117,151]. Summarizing, ballet-based interventions seem to be particularly beneficial for individuals with PD experiencing postural instability, axial rigidity, and reduced movement amplitude, especially when delivered in an adapted and supportive format [90,117,151,152].

#### 3.2.5. Contemporary Dance

Contemporary dance is characterized by expressive freedom, movement variability, and creativity rather than fixed technique. Unlike more structured dance forms, this dance style encourages exploration of space, weight, and personal movement expression [67,68,92,106,152]. Biomechanically, contemporary dance involves fluid transitions, floor-to-standing movements, and multidirectional motion, hence supporting mobility, dynamic balance, and adaptability to changing movement demands. The absence of rigid step patterns allows movement complexity to be tailored to the subjective functional capacity [91,92,152]. Cognitively, contemporary dance promotes exploratory motor planning, attentional flexibility, and problem-solving rather than memorization of predefined sequences. This open-ended structure seems to enhance engagement and reduce performance pressure, particularly for individuals with fluctuating motor symptoms [29,67,92,115]. Psychosocially, contemporary dance emphasizes emotional expression, embodiment, and identity reconstruction, thus addressing common PD-related psychosocial challenges, including loss of self-image and emotional distress [68,91,92,116]. Overall, contemporary dance appears particularly suitable to counteract non-motor symptoms and increase psychosocial wellbeing. In addition, it also promotes mobility and movement confidence through adaptive and participant-centered exploration [65,67,68,92].

#### 3.2.6. Folk and Traditional Dance

Folk dance is a broad term referred to the traditional dances of a community or a geographical area, typically passed down through generations, entailing a variegated pool of styles closely connected to local heritage and identity [134,153]. These styles are often rhythmically repetitive and characterized by simple step patterns performed in circles or lines, emphasizing collective movement rather than individual performance [61,94,99,154,155,156]. Biomechanically, repetitive stepping, coordinated weight shifts, and group pacing are strongly emphasized, hence promoting gait endurance, postural stability, and rhythmic coordination [95,96,97,157]. Furthermore, the simplicity of movements makes folk dance accessible across a wide range of functional abilities. Cognitively, such variegated dance style places modest demands on memory and attention, reducing cognitive load while maintaining motor–rhythmic integration and procedural learning [61,63,94,154]. Psychosocially, folk dance strongly promotes social inclusion, cultural identity, and community belonging, consequently enhancing motivation, adherence, and overall quality of life [60,137,154,155,156]. In parallel, the collective nature of participation may reduce performance anxiety and enhance enjoyment, particularly in community-based settings. Briefly summarizing, folk and traditional dances may be especially effective for promoting participation, adherence, and social wellbeing, while providing moderate motor stimulation suitable for individuals at different PD stages [61,94,95,96,97,154,157].

#### 3.2.7. Urban Dance

Urban and street dance styles are characterized by dynamic movement patterns, rapid transitions, and rhythmic variability. Compared to other dance styles, these emphasize speed, coordination, and responsiveness to complex musical rhythms [158]. Biomechanically, urban dance involves quick directional changes, lower-limb agility, and whole-body coordination consequently challenging agility, reaction time, dynamic balance, and coordinated weight shifting. Overall, such features potentially support motor adaptability in individuals with preserved functional capacity [98,158]. Cognitively, urban dance requires rapid processing of rhythmic cues, attentional control, and adaptive motor planning. Additionally, the energetic, percussive, and contemporary nature of music may further enhance arousal and engagement. Psychosocially, this variegated dance style may appeal particularly to younger individuals with PD, offering identity-affirming, socially engaging, and empowering movement experiences [98]. Urban dance for PD-affected subjects has been almost no investigated. Nevertheless, a recent pilot trial focusing on popping, a hip hop derived style, highlighted acute mood benefits in this target population [98]. Specifically, a decrease in anxiety and depression symptoms along with an improvement in overall wellbeing and vitality have been detected after a single session. Such beneficial effects, though time-limited, seem to be inherently linked to the specific features of popping. Indeed, this urban dance style is characterized by syncopated percussive beats and controlled muscle contraction-release that seem to stimulate dopaminergic pathways and motor timing [98]. Additionally, the music-driven expressive and playful context provided by popping classes allows participants to experience a sense of mastery and autonomy, hence positively impacting self-esteem and mood. Although further rigorous research is needed, this pilot study suggests that sustained practice may potentially lead to promising long-term psychological benefits in these vulnerable individuals [98]. In general, urban dance may be best suited for individuals in earlier PD stages who are capable of higher-intensity movement and who benefit from challenging, motivating, and culturally contemporary forms of engagement [98,158].

The aforementioned specific features of the different dance styles and their related benefits for PD are schematically summarized in Table 1.

#### 3.2.8. Comparative Synthesis of Evidence

Despite the abovementioned dance styles share foundational therapeutic/re-educational mechanisms, their distinct combinations of motor demands, cognitive load, emotional engagement, and multisensory cueing suggest differential benefits and advantages. Partnered disciplines such as Argentine tango and ballroom dance strongly challenge dynamic balance, weight shifting, and adaptive responses to external cues, which are particularly relevant for freezing of gait and fall prevention [20,23,24,60,87,99,142]. In contrast, non-partnered or group-based styles also yield meaningful motor improvements, suggesting that external rhythmic structure rather than partner contact alone is a key therapeutic driver [67,94,117]. Specifically, ballet and contemporary dance emphasize internally guided movement, postural alignment, and body awareness, potentially addressing axial rigidity, balance confidence, and motor planning deficits [67,68,88,91,117,151]. These internally driven strategies are relevant for impairments in motor initiation and proprioception commonly observed in PD [14,17,92,120,151]. Although broadly comparable efficacy for core motor outcomes has been demonstrated across different dance styles, their comparative advantages appear to lie in qualitative dimensions such as emotional resonance, social meaning, sensory richness, and cognitive demand rather than in isolated biomechanical effects [60,68,94,116,144]. Structured dances with predefined step patterns (e.g., tango, ballroom, and ballet) emphasize sequencing, working memory, and anticipatory planning, which may preferentially support executive function and dual-task performance [97,99,117,145,151]. Conversely, contemporary, improvisational, and creative dance forms place greater demands on cognitive flexibility, action selection, and sensorimotor exploration, potentially fostering adaptive motor strategies and creativity [67,116,161]. On this basis, different styles may offer partially overlapping but complementary cognitive training profiles rather than uniform cognitive effects [67,68,71]. Folk and social dance forms integrate rhythmic stepping with group participation, enhancing enjoyment, motivation, and adherence, which are key determinants of long-term exercise engagement in PD [47,48,59,60,94,137]. Evidence from these modalities highlighted benefits extending beyond motor function to mood, social participation, and quality of life, thus underscoring the importance of affective and social dimensions of rehabilitation [94,96,97,155,157].

In summary, the therapeutic value of dance-based interventions in PD is mechanism-dependent rather than style-exclusive, arising from the dynamic interaction between rhythmic cueing, motor complexity, cognitive engagement, and emotional–social meaning [69,71,119,133]. Acknowledging both shared foundations and style-specific features might effectively enable a more tailored selection of dance-based interventions adapted to symptom profile, disease stage, and subjective preference. Such evidence-based approach might help fostering and spreading the rationale for dance-based programs as a personalized and integrative therapy in PD [32,51,68,99,119,130].

## 4. Discussion and Future Directions

Literature synthesized in this narrative review supports dance-based interventions as a uniquely patient-centered approach to effectively address the multidimensional complex spectrum of PD-related motor, cognitive, and psychosocial symptoms and challenges [51,61,62,64,162]. Indeed, research repeatedly highlighted short-term functional improvements in sensorimotor domains such as postural stability, balance, gait dynamics, and coordination [32,33,55,150]. Simultaneously, even cognitive performance, attention, mood, and social bonding resulted enhanced after participating in dance classes [31,57,58,62,77]. Collectively, such evidence suggests that dance-based programs may effectively target PD-related deficits by globally engaging practitioners through specific mechanisms that extend far beyond conventional physical activity [13,51,60,143,163]. However, they are still under investigated. A central hypothesis emerging from our literature overview directly links the aforementioned benefits to the inherent integration of rhythmic cueing, coordinative motor tasks, cognitive engagement, emotional expression, and social interaction generally characterizing dance [2,78,103,134]. Owing to these features, multiple neural systems notoriously compromised in PD are stimulated, hence fostering neuroplasticity and recruitment of compensatory circuits [104,108,150]. From a neurophysiological perspective, motor impairments in PD are linked to dysfunctional cortico–basal ganglia circuits, especially excessive beta-band oscillations that disrupt movement initiation and timing [2,14,164]. Dance-based interventions, through rhythmic auditory–motor coupling, may modulate these oscillations and engage alternative timing networks, including premotor, supplementary motor, and cerebellar regions [51,104,138]. In particular, rhythmic entrainment, a common element investigated across studies on different dance styles, emerged to play a crucial role in bypassing basal ganglia dysfunction by promoting externally cued movement initiation, sequencing, and regulation [55,72,79,100,104,106,108,109,114]. Indeed, rhythmic entrainment functions not only as an external cue but also as a neuromodulatory stimulus, providing predictable temporal structure that enhances sensorimotor integration and reduces reliance on impaired internal cueing [110,113]. Moreover, the motor–cognitive demands arising during dance practice, peculiarly choreographic sequences memorization, visuospatial and attentional control, movement adaptation to partner/group/rhythm, and improvisational decision-making, significantly challenge executive function and motor planning [57,62,120,121]. In detail, such multimodal demands activate fronto-striatal and cortico-cerebellar networks involved in executive control, working memory, and motor planning, supporting top-down facilitation of movement and compensating for impaired automaticity [13,87]. These network-level effects likely underpin improvements in dual-task performance and functional adaptability observed across dance interventions [55,111]. Therefore, dance-based intervention for PD-affected individuals may potentially mitigate psychophysical decline while promoting flexible motor–cognitive strategies that benefit real-world functional adaptability [64,143,165].

The analyzed evidence accurately documented and quantitatively assessed sensorimotor benefits of dance-based interventions for PD. Conversely, cognitive, emotional, and social processes underlying improvements in non-motor symptomatology have been underexamined [68,162,166]. The intrinsic expressive, communal, and non-medicalized context of dance classes offers concrete opportunities for emotional release, interpersonal bonding, and identity reconstruction following PD diagnosis [66,88,125,129]. The contribution of these psychosocial factors to long-term adherence and functional outcomes can be conceptually framed recalling the established behavioral model known as Self-Determination Theory [167,168]. Dance-based interventions inherently support autonomy through creative and expressive movement choices, competence through progressive mastery of motor tasks, and relatedness through partner and group interaction [13]. By satisfying these needs, dance might foster intrinsic motivation which is a critical determinant of sustained engagement in physical activity, especially in chronic neurodegenerative conditions such as PD [51,59,110]. Furthermore, enhanced self-efficacy emerging from successful movement experiences and social affirmation may mediate the transfer of gains from the dance setting to daily life activities, thereby reinforcing functional independence, participation, and quality of life [13,56,64]. Taken together, such beneficial effects deeply influence long-term adherence to practice, a well-documented barrier in conventional exercise interventions addressing PD-affected individuals [51,57,136]. Therefore, these key psychosocial aspects need to be rigorously investigated and deepened. Likewise, only a few studies explored neurophysiological changes in response to dance in this target population, thus highlighting a lack of research focused on the potential contribution of dance-based interventions to neuroprotection and slowing functional decline [64,69]. Although direct evidence is limited, complex activities like dance have been associated with reduced neuroinflammation, lower oxidative stress, and upregulation of neurotrophic factors such as brain-derived neurotrophic factor, which supports synaptic plasticity and neuronal survival [44,45]. The multimodal nature of dance, combining aerobic effort, cognitive, emotional, and social engagement, may synergistically modulate dopaminergic signaling, stress hormones, and limbic system activation [59,113,131]. Such combined stimulation might potentially enhance neural resilience and slow functional decline, though targeted mechanistic studies specifically addressing dance-related neuroprotection in PD are still needed [114,163]. While early evidence investigating long-term practice demonstrated that prolonged engagement in dance-based activities may influence the disease progression, it remains unclear whether these effects derive from specific parameters such as rhythmic cueing, social interaction, and intensity, or their complex interplay [69]. Our literature analysis evidenced a broad heterogeneity in the investigated samples (e.g., number of enrolled participants, sociodemographic features, and disease stage), protocol characteristics (e.g., duration, intensity, frequency, and setting), dance styles, and primary outcome measures [63,67,162]. Despite it is well-known that sensorimotor and psychosocial outcomes are strongly influenced by intervention features, only a small body of literature rigorously isolated components such as cuing modality, partnering, or improvisational tasks. Consequently, cross-study comparison and identification of consistent dose–response relationship become particularly challenging [69,98,130]. Clarifying and deepening these critical issues become essential to optimize the practical and stable implementation of dance-based interventions within the variegated spectrum of PD management settings. Nevertheless, given the complex and multidimensional nature of dance as an artistic, psychophysical, cognitive, and socioemotional discipline [2,53,78,169,170], the isolation of specific dimensions/mechanisms is inherently difficult [13,53,67].

From a methodological perspective, the major gap identified across studies is attributable to an overall lack of randomized controlled trials and control groups, risk of bias, the predominance of a few research groups, and the scarce comparison of specific effects of the different styles [51,64,68,105]. Specifically, small sample sizes, often under 30 participants, reduced statistical power and generalizability [64,99,150]. Randomized controlled trials were relatively scarce, with most studies using pilot, single-group, or quasi-experimental designs [87,140]. Risks of bias mainly arise from limited allocation concealment, unblinded outcome assessment, and incomplete reporting of adherence [76]. Gaps in detailing intervention intensity, instructor qualifications, and protocol fidelity further hindered reproducibility and cross-study comparison [99,114]. Additionally, only few studies included active comparators, such as conventional exercise or social activities, hence complicating isolation of dance-specific effects [72,150]. Due to such study design weaknesses, the transferability of the previously detailed promising findings and their on-field application are often hindered. While methodological limitations remain and require systematic attention, literature is increasingly supporting the practical value of incorporating dance-based interventions into multidisciplinary PD management [53,77,100]. By providing physical, psychological, and socioemotional accessible challenges, and offering a wide range of styles/rhythms, dance represents an accessible, adaptable, and enjoyable activity particularly aligned with the contemporary models of integrative care [4,33,53].

Future research in this field should adopt a more structured and programmatic approach. In particular, adequately powered randomized controlled trials investigating style-specific effects, as well as dose–response relationships concerning intervention frequency, intensity, and duration should be prioritized [51,68,69]. Mechanistic studies employing objective evaluative tools, such as motion capture, wearable sensors, neuroimaging, and electrophysiological measures, are needed to elucidate neural, biomechanical, and behavioral pathways underlying the observed benefits [68,162,171,172]. In parallel, further research should explore scalable community-based models, integration within multidisciplinary care pathways, and strategies to enhance accessibility across variegated socioeconomic and cultural contexts [33,173]. Evaluating caregiver involvement, long-term sustainability, and cost-effectiveness becomes essential to support the translation of dance-based interventions from research settings to real-world PD care [125,132,174]. Collaborative partnerships among healthcare providers, dance educators, and community organizations might be planned and supported to maximize real-world impact and sustainability of dance-based interventions [25,65]. Safety protocols, including gradual progression of task difficulty, environmental adaptations, partner/assistive support when needed, and monitoring of fatigue, are essential to ensure feasibility across disease stages. Regarding scalability, community-based dance programs offer a cost-effective and accessible alternative to clinical rehabilitation settings, though logistic/mobility barriers, socioeconomic constraints, cultural relevance, and territorial availability still frequently limit participation [47,48,175]. In addition, future studies should explicitly examine the role of instructor/teacher expertise, including training in PD–specific motor and non-motor symptomatology, use of multisensory cueing and adaptive teaching strategies, risk management, and the competence to individualize progression and task complexity. In line with such multidisciplinary complex needs and taking into account the psychophysical effort required by dance, specialized kinesiologists should be stably involved within the global management of this vulnerable target of individuals [176]. Indeed, their on-field trained competences might critically and positively influence safety, engagement, adherence, and therapeutic efficacy of dance-based interventions for PD [59,77,123,161].

## 5. Conclusions

In conclusion, growing evidence highlight that dance is progressively transitioning from a complementary recreational activity to a reliable and structured therapeutic intervention within the mainstream care models [33,53]. Dance, as a psychophysical and expressive engaging practice, uniquely supports the patient-centered holistic perspective on which integrative medicine is rooted. Therefore, its systematic and stable integration into multidisciplinary PD care perfectly meets the modern definition of health [177], indeed enabling these vulnerable individuals not only to merely cope with disease but primarily to optimize and regain independence, resilience, and quality of life.

## Figures and Tables

**Table 1:** Main investigated dance styles and evidence-based benefits.

Dance Style	Primary Investigated Outcomes	Main Reported Effects	Evidence-Based Interpretation	Main References
Argentine Tango	Motor function: gait, stride length, balance, turning, postural stability, and PD-specific motor scales. Cognitive outcomes: spatial cognition, executive function, and attention. Psychosocial outcomes: mood, quality of life, and fatigue. Feasibility, safety, and adherence.	Consistent improvements in gait dynamics/parameters, rhythmic coordination, balance, postural stability, and functional mobility. Reduced fall risk. Cognitive-motor benefits (visuospatial cognition, motor planning, and executive function). Enhanced mood and quality of life. High feasibility, adherence, and enjoyment.	Moderate-strong evidence for improving motor and non-motor domains. Benefits due to combination of rhythmic cueing, partner interaction, cognitive engagement, and dynamic balance challenges. Highly promising and feasible movement-based therapy for PD.	[79,80,81,82,83,87,114,118,131,139,141,142,143,145,146,147,159,160]
Ballroom Dance	Motor function: balance, gait speed, stride length, mobility, and PD-specific motor scales. Cognitive outcomes: executive function and visuospatial skills. Psychosocial outcomes: quality of life, mood, and emotional regulation.	Consistent improvements in balance with moderate-strong gains in gait and functional mobility. Enhanced quality of life and positive effects on mood and emotional wellbeing. Cognitive benefits (executive function and visuospatial cognition).	Reliable benefits in balance, gait, and quality of life for individuals with PD. Motor and psychosocial benefits due to variegated rhythm/styles engagement, coordinative/cognitive demands, and motor–cognitive integration. Feasible and clinically valuable movement-based therapy for PD.	[85,86,87,100,136,148,149]
Classical Ballet and Ballet Techniques	Motor function: gait/gait variability, balance, postural control, mobility, freezing of gait, and strength. Cognitive outcomes: attention, executive function, and cue-responsiveness. Psychosocial outcomes: psychological and emotional wellbeing. Feasibility and acceptability.	Motor benefits, reduced gait variability, improved balance confidence, postural alignment, and weight-shift control. Improved mobility in individuals experiencing freezing of gait. Psychosocial benefits, enhanced confidence, wellbeing, and social connection. High adherence, enjoyment, and feasibility.	External cues and rhythmic guidance help bypass impaired internal motor generation, especially freezing of gait. Emphasis on postural alignment and controlled weight transfer improve gait consistency and perceived stability. Feasible and clinically valuable movement-based therapy for PD.	[88,90,117,151,152]
Contemporary Dance	Motor function: gait, postural control, balance, and functional mobility. Cognitive outcomes: attention, executive function, and cue-responsiveness. Psychosocial outcomes: psychological and emotional wellbeing, quality of life. Feasibility and acceptability.	Improved gait, balance, postural control, and mobility. Enhanced self-perception, mood, psychosocial wellbeing, and quality of life. High feasibility and engagement.	External cues and rhythmic guidance help bypass impaired internal motor generation. Psychosocial benefits due to creative, expressive, and participant-driven approaches. Self-perception, motivation, and motor–cognitive improvements due to unconventional movements. Feasible and clinically valuable movement-based therapy for PD.	[65,67,91,92,152,161,162]
Folk and Traditional Dance	Motor function: gait, postural control, balance, and PD-specific motor scales. Cognitive outcomes: attention, executive function, and dual-task performance. Psychosocial outcomes: psychological and emotional wellbeing, quality of life, and social engagement. Feasibility and adherence.	Improved gait, balance, postural control, and functional mobility. Enhanced mood, social engagement, psychological wellbeing, and quality of life. High feasibility, engagement, and adherence.	External cues and rhythmic guidance help bypass impaired internal motor generation. Group-based/formation patterns and repetitive/predictable step sequences improve motor–cognitive integration and movement confidence. Psychosocial benefits due to multiple styles/rhythm, social engagement, and cultural relevance/adaptability. Feasible and clinically valuable movement-based therapy for PD.	[61,94,95,96,105,154,155,156,157]
Urban Dance (i.e., Popping)	Mood.	Short-term improved mood, decreased depression and confusion.	Rhythmic emphasis (syncopated percussive beats) stimulates dopaminergic pathways and motor timing. Mood benefits due to proprioceptive awareness stimulation, creative self-expression, and interactive/playful context.	[98]

PD, Parkinson’s disease.

## Data Availability

No new data were created or analyzed in this study. Data sharing is not applicable to this article.

## Abbreviations

The following abbreviations are used in this manuscript:

PD	Parkinson’s disease

## References

[1] Feigin V.L., Vos T., Nichols E., Owolabi M.O., Carroll W.M., Dichgans M., Deuschl G., Parmar P., Brainin M., Murray C. (2020). The Global Burden of Neurological Disorders: Translating Evidence into Policy. Lancet Neurol..

[2] Gronek P., Haas A.N., Czarny W., Podstawski R., do Santos Delabary M., Clark C.C., Boraczyński M., Tarnas M., Wycichowska P., Pawlaczyk M. (2021). The Mechanism of Physical Activity-Induced Amelioration of Parkinson’s Disease: A Narrative Review. Aging Dis..

[3] Savica R., Grossardt B.R., Rocca W.A., Bower J.H. (2018). Parkinson Disease with and without Dementia: A Prevalence Study and Future Projections. Mov. Disord..

[4] Hussain F., Farooqui S., Khan I.A., Hassan B., Afridi Z.K. (2023). Effects of Exercise-Based Management on Motor Symptoms in Parkinson’s Disease—A Meta-Analysis. J. Coll. Physicians Surg. Pak..

[5] Dorsey E.R., Sherer T., Okun M.S., Bloem B.R. (2018). The Emerging Evidence of the Parkinson Pandemic. J. Parkinson’s Dis..

[6] Reich S.G., Savitt J.M. (2019). Parkinson’s Disease. Med. Clin. N. Am..

[7] Armstrong M.J., Okun M.S. (2020). Diagnosis and Treatment of Parkinson Disease: A Review. JAMA.

[8] Hayes M.T. (2019). Parkinson’s Disease and Parkinsonism. Am. J. Med..

[9] Hidalgo-Agudo R.D., Lucena-Anton D., Luque-Moreno C., Heredia-Rizo A.M., Moral-Munoz J.A. (2020). Additional Physical Interventions to Conventional Physical Therapy in Parkinson’s Disease: A Systematic Review and Meta-Analysis of Randomized Clinical Trials. J. Clin. Med..

[10] Lorenzo-García P., Núñez de Arenas-Arroyo S., Cavero-Redondo I., Guzmán-Pavón M.J., Priego-Jiménez S., Álvarez-Bueno C. (2023). Physical Exercise Interventions on Quality of Life in Parkinson Disease: A Network Meta-Analysis. J. Neurol. Phys. Ther..

[11] Zhao N., Yang Y., Zhang L., Zhang Q., Balbuena L., Ungvari G.S., Zang Y.-F., Xiang Y.-T. (2021). Quality of Life in Parkinson’s Disease: A Systematic Review and Meta-Analysis of Comparative Studies. CNS Neurosci. Ther..

[12] van Munster E.P.J., van der Aa H.P.A., Verstraten P., Heymans M.W., van Nispen R.M.A. (2022). Improved Intention, Self-Efficacy and Social Influence in the Workspace May Help Low Vision Service Workers to Discuss Depression and Anxiety with Visually Impaired and Blind Adults. BMC Health Serv. Res..

[13] Carapellotti A.M., Meijerink H.J.E.M., Gravemaker-Scott C., Thielman L., Kool R., Lewin N., Abma T.A. (2023). Escape, Expand, Embrace: The Transformational Lived Experience of Rediscovering the Self and the Other While Dancing with Parkinson’s or Multiple Sclerosis. Int. J. Qual. Stud. Health Well-being.

[14] Moustafa A.A., Chakravarthy S., Phillips J.R., Gupta A., Keri S., Polner B., Frank M.J., Jahanshahi M. (2016). Motor Symptoms in Parkinson’s Disease: A Unified Framework. Neurosci. Biobehav. Rev..

[15] di Biase L., Summa S., Tosi J., Taffoni F., Marano M., Cascio Rizzo A., Vecchio F., Formica D., Di Lazzaro V., Di Pino G. (2018). Quantitative Analysis of Bradykinesia and Rigidity in Parkinson’s Disease. Front. Neurol..

[16] Hirakawa Y., Sakurai H., Takeda K., Koyama S., Iwai M., Motoya I., Kanada Y., Kawamura N., Kawamura M., Tanabe S. (2025). Relationship Between Each of the Four Major Motor Symptoms and At-Home Physical Activity in Individuals with Parkinson’s Disease: A Cross-Sectional Study. Neurol. Int..

[17] Pieruccini-Faria F., Jones J.A., Almeida Q.J. (2016). Insight into Dopamine-Dependent Planning Deficits in Parkinson’s Disease: A Sharing of Cognitive & Sensory Resources. Neuroscience.

[18] Tamilselvam Y.K., Jog M., Patel R.V. (2023). Robot-Assisted Investigation of Sensorimotor Control in Parkinson’s Disease. Sci. Rep..

[19] Falletti M., Asci F., Zampogna A., Patera M., Suppa A. (2025). Cogwheel Rigidity in Parkinson’s Disease: Clinical, Biomechanical and Neurophysiological Features. Neurobiol. Dis..

[20] Viseux F.J.F., Delval A., Defebvre L., Simoneau M. (2020). Postural Instability in Parkinson’s Disease: Review and Bottom-up Rehabilitative Approaches. Neurophysiol. Clin..

[21] Abusrair A.H., Elsekaily W., Bohlega S. (2022). Tremor in Parkinson’s Disease: From Pathophysiology to Advanced Therapies. Tremor Other Hyperkinet Mov..

[22] Boonstra T.A., van der Kooij H., Munneke M., Bloem B.R. (2008). Gait Disorders and Balance Disturbances in Parkinson’s Disease: Clinical Update and Pathophysiology. Curr. Opin. Neurol..

[23] Mirelman A., Bonato P., Camicioli R., Ellis T.D., Giladi N., Hamilton J.L., Hass C.J., Hausdorff J.M., Pelosin E., Almeida Q.J. (2019). Gait Impairments in Parkinson’s Disease. Lancet Neurol..

[24] Bloem B.R., Hausdorff J.M., Visser J.E., Giladi N. (2004). Falls and Freezing of Gait in Parkinson’s Disease: A Review of Two Interconnected, Episodic Phenomena. Mov. Disord..

[25] Leite Silva A.B.R., Gonçalves de Oliveira R.W., Diógenes G.P., de Castro Aguiar M.F., Sallem C.C., Lima M.P.P., de Albuquerque Filho L.B., Peixoto de Medeiros S.D., Penido de Mendonça L.L., de Santiago Filho P.C. (2023). Premotor, Nonmotor and Motor Symptoms of Parkinson’s Disease: A New Clinical State of the Art. Ageing Res. Rev..

[26] Opara J.A., Brola W., Leonardi M., Błaszczyk B. (2012). Quality of Life in Parkinson’s Disease. J. Med. Life.

[27] Marinus J., Ramaker C., van Hilten J.J., Stiggelbout A.M. (2002). Health Related Quality of Life in Parkinson’s Disease: A Systematic Review of Disease Specific Instruments. J. Neurol. Neurosurg. Psychiatry.

[28] Shin H.-W., Hong S.-W., Youn Y.C. (2022). Clinical Aspects of the Differential Diagnosis of Parkinson’s Disease and Parkinsonism. J. Clin. Neurol..

[29] Intzandt B., Beck E.N., Silveira C.R.A. (2018). The Effects of Exercise on Cognition and Gait in Parkinson’s Disease: A Scoping Review. Neurosci. Biobehav. Rev..

[30] LeWitt P.A. (2015). Levodopa Therapy for Parkinson’s Disease: Pharmacokinetics and Pharmacodynamics. Mov. Disord..

[31] Wang Y., Sun X., Li F., Li Q., Jin Y. (2022). Efficacy of Non-Pharmacological Interventions for Depression in Individuals with Parkinson’s Disease: A Systematic Review and Network Meta-Analysis. Front. Aging Neurosci..

[32] Barnish M.S., Reynolds S.E., Nelson-Horne R.V. (2025). Active Group-Based Performing Arts Interventions in Parkinson’s Disease: An Updated Systematic Review and Meta-Analysis. BMJ Open.

[33] Kola S., Subramanian I. (2023). Updates in Parkinson’s Disease Integrative Therapies: An Evidence-Based Review. Curr. Neurol. Neurosci. Rep..

[34] Šumec R., Filip P., Sheardová K., Bareš M. (2015). Psychological Benefits of Nonpharmacological Methods Aimed for Improving Balance in Parkinson’s Disease: A Systematic Review. Behav. Neurol..

[35] Bloem B.R., de Vries N.M., Ebersbach G. (2015). Nonpharmacological Treatments for Patients with Parkinson’s Disease. Mov. Disord..

[36] Tinazzi M., Abbruzzese G., Antonini A., Ceravolo R., Fabbrini G., Lessi P., Barone P., REASON Study Group (2013). Reasons Driving Treatment Modification in Parkinson’s Disease: Results from the Cross-Sectional Phase of the REASON Study. Parkinsonism Relat. Disord..

[37] Baker L.D., Frank L.L., Foster-Schubert K., Green P.S., Wilkinson C.W., McTiernan A., Plymate S.R., Fishel M.A., Watson G.S., Cholerton B.A. (2010). Effects of Aerobic Exercise on Mild Cognitive Impairment: A Controlled Trial. Arch. Neurol..

[38] Lorenzo-García P., Cavero-Redondo I., Núñez de Arenas-Arroyo S., Guzmán-Pavón M.J., Priego-Jiménez S., Álvarez-Bueno C. (2024). Effects of Physical Exercise Interventions on Balance, Postural Stability and General Mobility in Parkinson’s Disease: A Network Meta-Analysis. J. Rehabil. Med..

[39] Alberts J.L., Rosenfeldt A.B. (2020). The Universal Prescription for Parkinson’s Disease: Exercise. J. Parkinson’s Dis..

[40] Oguh O., Eisenstein A., Kwasny M., Simuni T. (2014). Back to the Basics: Regular Exercise Matters in Parkinson’s Disease: Results from the National Parkinson Foundation QII Registry Study. Parkinsonism Relat. Disord..

[41] Bhalsing K.S., Abbas M.M., Tan L.C.S. (2018). Role of Physical Activity in Parkinson’s Disease. Ann. Indian Acad. Neurol..

[42] Mak M.K., Wong-Yu I.S., Shen X., Chung C.L. (2017). Long-Term Effects of Exercise and Physical Therapy in People with Parkinson Disease. Nat. Rev. Neurol..

[43] Sabino-Carvalho J.L., Fisher J.P., Vianna L.C. (2021). Autonomic Function in Patients with Parkinson’s Disease: From Rest to Exercise. Front. Physiol..

[44] Kaagman D.G.M., van Wegen E.E.H., Cignetti N., Rothermel E., Vanbellingen T., Hirsch M.A. (2024). Effects and Mechanisms of Exercise on Brain-Derived Neurotrophic Factor (BDNF) Levels and Clinical Outcomes in People with Parkinson’s Disease: A Systematic Review and Meta-Analysis. Brain Sci..

[45] Mahalakshmi B., Maurya N., Lee S.-D., Bharath Kumar V. (2020). Possible Neuroprotective Mechanisms of Physical Exercise in Neurodegeneration. Int. J. Mol. Sci..

[46] Garcia Ruiz P.J., Luquin Piudo R., Martinez Castrillo J.C. (2022). On Disease Modifying and Neuroprotective Treatments for Parkinson’s Disease: Physical Exercise. Front. Neurol..

[47] Afshari M., Yang A., Bega D. (2017). Motivators and Barriers to Exercise in Parkinson’s Disease. J. Parkinson’s Dis..

[48] Schootemeijer S., van der Kolk N.M., Ellis T., Mirelman A., Nieuwboer A., Nieuwhof F., Schwarzschild M.A., de Vries N.M., Bloem B.R. (2020). Barriers and Motivators to Engage in Exercise for Persons with Parkinson’s Disease. J. Parkinson’s Dis..

[49] Cui W., Li D., Yue L., Xie J. (2023). The Effects of Exercise Dose on Patients with Parkinson’s Disease: A Systematic Review and Meta-Analysis of Randomized Controlled Trials. J. Neurol..

[50] Kim Y., Lai B., Mehta T., Thirumalai M., Padalabalanarayanan S., Rimmer J.H., Motl R.W. (2019). Exercise Training Guidelines for Multiple Sclerosis, Stroke, and Parkinson Disease: Rapid Review and Synthesis. Am. J. Phys. Med. Rehabil..

[51] Carapellotti A.M., Stevenson R., Doumas M. (2020). The Efficacy of Dance for Improving Motor Impairments, Non-Motor Symptoms, and Quality of Life in Parkinson’s Disease: A Systematic Review and Meta-Analysis. PLoS ONE.

[52] Colombo B., Rigby A., Gnerre M., Biassoni F. (2022). The Effects of a Dance and Music-Based Intervention on Parkinson’s Patients’ Well-Being: An Interview Study. Int. J. Environ. Res. Public Health.

[53] Cox L., Youmans-Jones J. (2023). Dance Is a Healing Art. Curr. Treat. Options Allergy.

[54] Earhart G.M. (2009). Dance as Therapy for Individuals with Parkinson Disease. Eur. J. Phys. Rehabil. Med..

[55] Haputhanthirige N.K.H., Sullivan K., Moyle G., Brauer S., Jeffrey E.R., Kerr G. (2023). Effects of Dance on Gait and Dual-Task Gait in Parkinson’s Disease. PLoS ONE.

[56] McNeely M.E., Duncan R.P., Earhart G.M. (2015). Impacts of Dance on Non-Motor Symptoms, Participation, and Quality of Life in Parkinson Disease and Healthy Older Adults. Maturitas.

[57] Fong Yan A., Cobley S., Chan C., Pappas E., Nicholson L.L., Ward R.E., Murdoch R.E., Gu Y., Trevor B.L., Vassallo A.J. (2018). The Effectiveness of Dance Interventions on Physical Health Outcomes Compared to Other Forms of Physical Activity: A Systematic Review and Meta-Analysis. Sports Med..

[58] Raje P., Ning S., Branson C., Saint-Hilaire M., de Leon M.P., DePold Hohler A. (2019). Self-Reported Exercise Trends in Parkinson’s Disease Patients. Complement. Ther. Med..

[59] Emmanouilidis S., Hackney M.E., Slade S.C., Heng H., Jazayeri D., Morris M.E. (2021). Dance Is an Accessible Physical Activity for People with Parkinson’s Disease. Parkinson’s Dis..

[60] Barnish M.S., Barran S.M. (2020). A Systematic Review of Active Group-Based Dance, Singing, Music Therapy and Theatrical Interventions for Quality of Life, Functional Communication, Speech, Motor Function and Cognitive Status in People with Parkinson’s Disease. BMC Neurol..

[61] Duarte J.D.S., Alcantara W.A., Brito J.S., Barbosa L.C.S., Machado I.P.R., Furtado V.K.T., Santos-Lobato B.L.D., Pinto D.S., Krejcová L.V., Bahia C.P. (2023). Physical Activity Based on Dance Movements as Complementary Therapy for Parkinson’s Disease: Effects on Movement, Executive Functions, Depressive Symptoms, and Quality of Life. PLoS ONE.

[62] Ismail S.R., Lee S.W.H., Merom D., Megat Kamaruddin P.S.N., Chong M.S., Ong T., Lai N.M. (2021). Evidence of Disease Severity, Cognitive and Physical Outcomes of Dance Interventions for Persons with Parkinson’s Disease: A Systematic Review and Meta-Analysis. BMC Geriatr..

[63] Bearss K.A., Barnstaple R.E., Bar R.J., DeSouza J.F.X. (2024). Impact of Weekly Community-Based Dance Training Over 8 Months on Depression and Blood Oxygen Level-Dependent Signals in the Subcallosal Cingulate Gyrus for People with Parkinson Disease: Observational Study. JMIRx Med..

[64] Bearss K.A., DeSouza J.F.X. (2021). Parkinson’s Disease Motor Symptom Progression Slowed with Multisensory Dance Learning over 3-Years: A Preliminary Longitudinal Investigation. Brain Sci..

[65] Carapellotti A.M., Rodger M., Doumas M. (2022). Evaluating the Effects of Dance on Motor Outcomes, Non-Motor Outcomes, and Quality of Life in People Living with Parkinson’s: A Feasibility Study. Pilot Feasibility Stud..

[66] Bognar S., DeFaria A.M., O’Dwyer C., Pankiw E., Simic Bogler J., Teixeira S., Nyhof-Young J., Evans C. (2017). More than Just Dancing: Experiences of People with Parkinson’s Disease in a Therapeutic Dance Program. Disabil. Rehabil..

[67] Bek J., Arakaki A.I., Derbyshire-Fox F., Ganapathy G., Sullivan M., Poliakoff E. (2022). More Than Movement: Exploring Motor Simulation, Creativity, and Function in Co-Developed Dance for Parkinson’s. Front. Psychol..

[68] Bek J., Arakaki A.I., Lawrence A., Sullivan M., Ganapathy G., Poliakoff E. (2020). Dance and Parkinson’s: A Review and Exploration of the Role of Cognitive Representations of Action. Neurosci. Biobehav. Rev..

[69] Devlin K., Alshaikh J.T., Pantelyat A. (2019). Music Therapy and Music-Based Interventions for Movement Disorders. Curr. Neurol. Neurosci. Rep..

[70] McNeely M.E., Mai M.M., Duncan R.P., Earhart G.M. (2015). Differential Effects of Tango Versus Dance for PD in Parkinson Disease. Front. Aging Neurosci..

[71] Moratelli J.A., Alexandre K.H., Boing L., Swarowsky A., Corrêa C.L., de Guimarães A.C.A. (2022). Dance Rhythms Improve Motor Symptoms in Individuals with Parkinson’s Disease: A Randomized Clinical Trial. J. Dance Med. Sci..

[72] Hackney M.E., Earhart G.M. (2010). Effects of Dance on Gait and Balance in Parkinson’s Disease: A Comparison of Partnered and Nonpartnered Dance Movement. Neurorehabil. Neural Repair.

[73] Dos Santos Delabary M., Komeroski I.G., Monteiro E.P., Costa R.R., Haas A.N. (2018). Effects of Dance Practice on Functional Mobility, Motor Symptoms and Quality of Life in People with Parkinson’s Disease: A Systematic Review with Meta-Analysis. Aging Clin. Exp. Res..

[74] Gil P.R., Moratelli J.A., Fausto D.Y., Alexandre K.H., Garcia Meliani A.A., Lima A.G., Guimarães A.C.d.A. (2024). The Importance of Dance for the Cognitive Function of People with Parkinson’s: A Systematic Review with Meta-Analysis. J. Bodyw. Mov. Ther..

[75] Hasan S.M., Alshafie S., Hasabo E.A., Saleh M., Elnaiem W., Qasem A., Alzu’bi Y.O., Khaled A., Zaazouee M.S., Ragab K.M. (2022). Efficacy of Dance for Parkinson’s Disease: A Pooled Analysis of 372 Patients. J. Neurol..

[76] Heiberger L., Maurer C., Amtage F., Mendez-Balbuena I., Schulte-Mönting J., Hepp-Reymond M.-C., Kristeva R. (2011). Impact of a Weekly Dance Class on the Functional Mobility and on the Quality of Life of Individuals with Parkinson’s Disease. Front. Aging Neurosci..

[77] McGill A., Houston S., Lee R.Y.W. (2014). Dance for Parkinson’s: A New Framework for Research on Its Physical, Mental, Emotional, and Social Benefits. Complement. Ther. Med..

[78] Carretti G., Mirandola D., Sgambati E., Manetti M., Marini M. (2022). Survey on Psychological Well-Being and Quality of Life in Visually Impaired Individuals: Dancesport vs. Other Sound Input-Based Sports. Int. J. Environ. Res. Public Health.

[79] Hackney M.E., McKee K. (2014). Community-Based Adapted Tango Dancing for Individuals with Parkinson’s Disease and Older Adults. J. Vis. Exp..

[80] Rios Romenets S., Anang J., Fereshtehnejad S.-M., Pelletier A., Postuma R. (2015). Tango for Treatment of Motor and Non-Motor Manifestations in Parkinson’s Disease: A Randomized Control Study. Complement. Ther. Med..

[81] Giorgi F., Platano D., Berti L., Donati D., Tedeschi R. (2025). Dancing Towards Stability: The Therapeutic Potential of Argentine Tango for Balance and Mobility in Parkinson’s Disease. Diseases.

[82] Blandy L.M., Beevers W.A., Fitzmaurice K., Morris M.E. (2015). Therapeutic Argentine Tango Dancing for People with Mild Parkinson’s Disease: A Feasibility Study. Front. Neurol..

[83] Holmes W.M., Hackney M.E. (2017). Adapted Tango for Adults with Parkinson’s Disease: A Qualitative Study. Adapt. Phys. Activ. Q..

[84] Lötzke D., Ostermann T., Büssing A. (2015). Argentine Tango in Parkinson Disease--a Systematic Review and Meta-Analysis. BMC Neurol..

[85] Ashburn A., Roberts L., Pickering R., Roberts H.C., Wiles R., Kunkel D., Hulbert S., Robison J., Fitton C. (2014). A Design to Investigate the Feasibility and Effects of Partnered Ballroom Dancing on People with Parkinson Disease: Randomized Controlled Trial Protocol. JMIR Res. Protoc..

[86] Kunkel D., Fitton C., Roberts L., Pickering R.M., Roberts H.C., Wiles R., Hulbert S., Robison J., Ashburn A. (2017). A Randomized Controlled Feasibility Trial Exploring Partnered Ballroom Dancing for People with Parkinson’s Disease. Clin. Rehabil..

[87] Hackney M.E., Earhart G.M. (2009). Effects of Dance on Movement Control in Parkinson’s Disease: A Comparison of Argentine Tango and American Ballroom. J. Rehabil. Med..

[88] Houston S., McGill A. (2013). A Mixed-Methods Study into Ballet for People Living with Parkinson’s. Arts Health.

[89] Haussler A.M., Tueth L.E., Earhart G.M. (2025). Feasibility of a Barre Exercise Intervention for Individuals with Mild to Moderate Parkinson Disease. J. Dance Med. Sci..

[90] Podlewska A.M., Batzu L., Soukup T., Sevdalis N., Bakolis I., Derbyshire-Fox F., Hartley A., Healey A., Woods A., Crane N. (2024). The PD-Ballet Study: Study Protocol for a Randomised Controlled Single-Blind Hybrid Type 2 Clinical Trial Evaluating the Effects of Ballet Dancing on Motor and Non-Motor Symptoms in Parkinson’s Disease. BMC Complement. Med. Ther..

[91] Valverde-Guijarro E., Alguacil-Diego I.M., Vela-Desojo L., Cano-de-la-Cuerda R. (2022). Effects of Contemporary Dance and Physiotherapy Intervention on Balance and Postural Control in Parkinson’s Disease. Disabil. Rehabil..

[92] Bar A., Czamanski-Cohen J., Federman J.D. (2021). I Feel Like I Am Flying and Full of Life: Contemporary Dance for Parkinson’s Patients. Front. Psychol..

[93] Harrison E.C., Earhart G.M., Leventhal D., Quinn L., Mazzoni P. (2020). A Walking Dance to Improve Gait Speed for People with Parkinson Disease: A Pilot Study. Neurodegener. Dis. Manag..

[94] Solla P., Cugusi L., Bertoli M., Cereatti A., Della Croce U., Pani D., Fadda L., Cannas A., Marrosu F., Defazio G. (2019). Sardinian Folk Dance for Individuals with Parkinson’s Disease: A Randomized Controlled Pilot Trial. J. Altern. Complement. Med..

[95] Shanahan J., Bhriain O.N., Morris M.E., Volpe D., Clifford A.M. (2016). Irish Set Dancing Classes for People with Parkinson’s Disease: The Needs of Participants and Dance Teachers. Complement. Ther. Med..

[96] Shanahan J., Morris M.E., Bhriain O.N., Volpe D., Lynch T., Clifford A.M. (2017). Dancing for Parkinson Disease: A Randomized Trial of Irish Set Dancing Compared with Usual Care. Arch. Phys. Med. Rehabil..

[97] Volpe D., Signorini M., Marchetto A., Lynch T., Morris M.E. (2013). A Comparison of Irish Set Dancing and Exercises for People with Parkinson’s Disease: A Phase II Feasibility Study. BMC Geriatr..

[98] Sistarelli S., Annett L.E., Lovatt P.J. (2023). Effects of Popping for Parkinson’s Dance Class on the Mood of People with Parkinson’s Disease. Int. J. Ther. Rehabil..

[99] Sharp K., Hewitt J. (2014). Dance as an Intervention for People with Parkinson’s Disease: A Systematic Review and Meta-Analysis. Neurosci. Biobehav. Rev..

[100] Rocha P.A., Slade S.C., McClelland J., Morris M.E. (2017). Dance Is More than Therapy: Qualitative Analysis on Therapeutic Dancing Classes for Parkinson’s. Complement. Ther. Med..

[101] Duncan R.P., Earhart G.M. (2012). Randomized Controlled Trial of Community-Based Dancing to Modify Disease Progression in Parkinson Disease. Neurorehabil. Neural Repair.

[102] Gates P., Discenzo F.M., Kim J.H., Lemke Z., Meggitt J., Ridgel A.L. (2022). Analysis of Movement Entropy during Community Dance Programs for People with Parkinson’s Disease and Older Adults: A Cohort Study. Int. J. Environ. Res. Public Health.

[103] Simon J.R., Bek J., Ghanai K., Bearss K.A., Barnstaple R.E., Bar R.J., DeSouza J.F.X. (2024). Neural Effects of Multisensory Dance Training in Parkinson’s Disease: Evidence from a Longitudinal Neuroimaging Single Case Study. Front. Aging Neurosci..

[104] Thaut M.H., Kenyon G.P., Schauer M.L., McIntosh G.C. (1999). The Connection between Rhythmicity and Brain Function. IEEE Eng. Med. Biol. Mag..

[105] Volpe D., Baldassarre M.G., Bakdounes L., Campo M.C., Ferrazzoli D., Ortelli P. (2025). “Dance Well”-A Multisensory Artistic Dance Intervention for People with Parkinson’s Disease: A Pilot Study. Brain Sci..

[106] Allen J.L., McKay J.L., Sawers A., Hackney M.E., Ting L.H. (2017). Increased Neuromuscular Consistency in Gait and Balance after Partnered, Dance-Based Rehabilitation in Parkinson’s Disease. J. Neurophysiol..

[107] Pereira A.P.S., Marinho V., Gupta D., Magalhães F., Ayres C., Teixeira S. (2019). Music Therapy and Dance as Gait Rehabilitation in Patients with Parkinson Disease: A Review of Evidence. J. Geriatr. Psychiatry Neurol..

[108] Hashimoto H., Takabatake S., Miyaguchi H., Nakanishi H., Naitou Y. (2015). Effects of Dance on Motor Functions, Cognitive Functions, and Mental Symptoms of Parkinson’s Disease: A Quasi-Randomized Pilot Trial. Complement. Ther. Med..

[109] Ventura M.I., Barnes D.E., Ross J.M., Lanni K.E., Sigvardt K.A., Disbrow E.A. (2016). A Pilot Study to Evaluate Multi-Dimensional Effects of Dance for People with Parkinson’s Disease. Contemp. Clin. Trials.

[110] Ashoori A., Eagleman D.M., Jankovic J. (2015). Effects of Auditory Rhythm and Music on Gait Disturbances in Parkinson’s Disease. Front. Neurol..

[111] Hulbert S., Ashburn A., Roberts L., Verheyden G. (2017). Dance for Parkinson’s-The Effects on Whole Body Co-Ordination during Turning Around. Complement. Ther. Med..

[112] Mahmoud H.M., Al-Turkistani Z.I., Alayat M.S., Abd El-Kafy E.M., El Fiky A.A.R. (2023). Effect of Dancing on Freezing of Gait in Patients with Parkinson’s Disease: A Systematic Review and Meta-Analysis. NeuroRehabilitation.

[113] Krotinger A., Loui P. (2021). Rhythm and Groove as Cognitive Mechanisms of Dance Intervention in Parkinson’s Disease. PLoS ONE.

[114] Kalyani H.H.N., Sullivan K., Moyle G., Brauer S., Jeffrey E.R., Roeder L., Berndt S., Kerr G. (2019). Effects of Dance on Gait, Cognition, and Dual-Tasking in Parkinson’s Disease: A Systematic Review and Meta-Analysis. J. Parkinson’s Dis..

[115] Camicioli R., Morris M.E., Pieruccini-Faria F., Montero-Odasso M., Son S., Buzaglo D., Hausdorff J.M., Nieuwboer A. (2023). Prevention of Falls in Parkinson’s Disease: Guidelines and Gaps. Mov. Disord. Clin. Pract..

[116] Andreasson I., Björkdahl A., Fristedt S., Bergman P., Filipowicz K., Johansson I.-K., Santos Tavares Silva I. (2024). Dance for Parkinson, Multifaceted Experiences of Persons Living with Parkinson’s Disease. Scand. J. Occup. Ther..

[117] McGill A., Houston S., Lee R.Y.W. (2019). Effects of a Ballet-Based Dance Intervention on Gait Variability and Balance Confidence of People with Parkinson’s. Arts Health.

[118] Koch S.C., Mergheim K., Raeke J., Machado C.B., Riegner E., Nolden J., Diermayr G., von Moreau D., Hillecke T.K. (2016). The Embodied Self in Parkinson’s Disease: Feasibility of a Single Tango Intervention for Assessing Changes in Psychological Health Outcomes and Aesthetic Experience. Front. Neurosci..

[119] Shanahan J., Morris M.E., Bhriain O.N., Saunders J., Clifford A.M. (2015). Dance for People with Parkinson Disease: What Is the Evidence Telling Us?. Arch. Phys. Med. Rehabil..

[120] Mezzarobba S., Bonassi G., Avanzino L., Pelosin E. (2024). Action Observation and Motor Imagery as a Treatment in Patients with Parkinson’s Disease. J. Parkinson’s Dis..

[121] Zhang Q., Hu J., Wei L., Jia Y., Jin Y. (2019). Effects of Dance Therapy on Cognitive and Mood Symptoms in People with Parkinson’s Disease: A Systematic Review and Meta-Analysis. Complement. Ther. Clin. Pract..

[122] Lewis C., Annett L.E., Davenport S., Hall A.A., Lovatt P. (2016). Mood Changes Following Social Dance Sessions in People with Parkinson’s Disease. J. Health Psychol..

[123] Magrath J., Kenny S.J., Ingstrup M.S., Morrison L., Paglione V., McDonough M.H., Din C. (2025). “We’re All Here to Be Dancers Together”: Perspectives on Facilitating Dance Classes for Individuals with Parkinson’s. Adapt. Phys. Activ. Q..

[124] Stupacher J., Matthews T.E., Pando-Naude V., Foster Vander Elst O., Vuust P. (2022). The Sweet Spot between Predictability and Surprise: Musical Groove in Brain, Body, and Social Interactions. Front. Psychol..

[125] Sundström M., Jola C. (2021). “I’m Never Going to Be in Phantom of the Opera”: Relational and Emotional Wellbeing of Parkinson’s Carers and Their Partners in and Beyond Dancing. Front. Psychol..

[126] Westheimer O., McRae C., Henchcliffe C., Fesharaki A., Glazman S., Ene H., Bodis-Wollner I. (2015). Dance for PD: A Preliminary Investigation of Effects on Motor Function and Quality of Life among Persons with Parkinson’s Disease (PD). J. Neural Transm..

[127] Carroll S.J., Dale M.J., Bail K. (2023). “Out and Proud…. in All Your Shaking Glory” the Wellbeing Impact of a Dance Program with Public Dance Performance for People with Parkinson’s Disease: A Qualitative Study. Disabil. Rehabil..

[128] Cheng W.-H., Quan Y., Thompson W.F. (2024). The Effect of Dance on Mental Health and Quality of Life of People with Parkinson’s Disease: A Systematic Review and Three-Level Meta-Analysis. Arch. Gerontol. Geriatr..

[129] Gibson R., Robichaud S. (2020). Evaluating Dancing with Parkinson’s: Reflections from the Perspective of a Community Organization. Eval. Program. Plann..

[130] Vaseinasrabadi R., DeSouza J. (2025). Dancing through the Darkness: A Systematic Review of Dance as a Multidimensional Therapy for Parkinson’s Disease. Neurodegener. Dis. Manag..

[131] Foster E.R., Golden L., Duncan R.P., Earhart G.M. (2013). Community-Based Argentine Tango Dance Program Is Associated with Increased Activity Participation among Individuals with Parkinson’s Disease. Arch. Phys. Med. Rehabil..

[132] Prieto L., Norris M.L., Columna L. (2021). “Keep Moving”: Experiences of People with Parkinson’s and Their Care Partners in a Dance Class. Adapt. Phys. Activ. Q..

[133] Senter M., Ni Bhriain O., Clifford A.M. (2025). “You Need to Know That You Are Not Alone”: The Sustainability of Community-Based Dance Programs for People Living with Parkinson’s Disease. Disabil. Rehabil..

[134] Fitch W.T., Barnstaple R. (2024). Dance as Mindful Movement: A Perspective from Motor Learning and Predictive Coding. BMC Neurosci..

[135] Gharpure V., Parab S., Ryain A., Ghosh A. (2024). Therapeutic Potential of Recreation on Non-Motor Symptoms in Parkinson’s Disease: A Literature Review. Adv. Mind Body Med..

[136] Kunkel D., Robison J., Fitton C., Hulbert S., Roberts L., Wiles R., Pickering R., Roberts H., Ashburn A. (2018). It Takes Two: The Influence of Dance Partners on the Perceived Enjoyment and Benefits during Participation in Partnered Ballroom Dance Classes for People with Parkinson’s. Disabil. Rehabil..

[137] Gyrling T., Ljunggren M., Karlsson S. (2021). The Impact of Dance Activities on the Health of Persons with Parkinson’s Disease in Sweden. Int. J. Qual. Stud. Health Well-being.

[138] Nombela C., Hughes L.E., Owen A.M., Grahn J.A. (2013). Into the Groove: Can Rhythm Influence Parkinson’s Disease?. Neurosci. Biobehav. Rev..

[139] Hackney M.E., Kantorovich S., Levin R., Earhart G.M. (2007). Effects of Tango on Functional Mobility in Parkinson’s Disease: A Preliminary Study. J. Neurol. Phys. Ther..

[140] de Dreu M.J., Kwakkel G., van Wegen E.E.H. (2015). Partnered Dancing to Improve Mobility for People with Parkinson’s Disease. Front. Neurosci..

[141] Docu Axelerad A., Stroe A.Z., Muja L.F., Docu Axelerad S., Chita D.S., Frecus C.E., Mihai C.M. (2022). Benefits of Tango Therapy in Alleviating the Motor and Non-Motor Symptoms of Parkinson’s Disease Patients—A Narrative Review. Brain Sci..

[142] Peter S., Crock N.D., Billings B.J., Wu R., Sterling S., Koul S., Taber W.F., Pique K., Golan R., Maitland G. (2020). Argentine Tango Reduces Fall Risk in Parkinson’s Patients. J. Am. Med. Dir. Assoc..

[143] Rawson K.S., McNeely M.E., Duncan R.P., Pickett K.A., Perlmutter J.S., Earhart G.M. (2019). Exercise and Parkinson Disease: Comparing Tango, Treadmill, and Stretching. J. Neurol. Phys. Ther..

[144] de Natale E.R., Paulus K.S., Aiello E., Sanna B., Manca A., Sotgiu G., Leali P.T., Deriu F. (2017). Dance Therapy Improves Motor and Cognitive Functions in Patients with Parkinson’s Disease. NeuroRehabilitation.

[145] Rabini G., Meli C., Prodomi G., Speranza C., Anzini F., Funghi G., Pierotti E., Saviola F., Fumagalli G.G., Di Giacopo R. (2024). Tango and Physiotherapy Interventions in Parkinson’s Disease: A Pilot Study on Efficacy Outcomes on Motor and Cognitive Skills. Sci. Rep..

[146] Poier D., Rodrigues Recchia D., Ostermann T., Büssing A. (2019). A Randomized Controlled Trial to Investigate the Impact of Tango Argentino versus Tai Chi on Quality of Life in Patients with Parkinson Disease: A Short Report. Complement. Med. Res..

[147] Rabinovich D.B., Garretto N.S., Arakaki T., DeSouza J.F. (2021). A High Dose Tango Intervention for People with Parkinson’s Disease (PwPD). Adv. Integr. Med..

[148] Kalyani H.H., Sullivan K.A., Moyle G.M., Brauer S.G., Jeffrey E.R., Kerr G.K. (2020). Dance Improves Symptoms, Functional Mobility and Fine Manual Dexterity in People with Parkinson Disease: A Quasi-Experimental Controlled Efficacy Study. Eur. J. Phys. Rehabil. Med..

[149] Wu C.-C., Xiong H.-Y., Zheng J.-J., Wang X.-Q. (2022). Dance Movement Therapy for Neurodegenerative Diseases: A Systematic Review. Front. Aging Neurosci..

[150] McNeely M.E., Duncan R.P., Earhart G.M. (2015). A Comparison of Dance Interventions in People with Parkinson Disease and Older Adults. Maturitas.

[151] Haussler A.M., Earhart G.M. (2023). The Effectiveness of Classical Ballet as a Therapeutic Intervention: A Narrative Review. Altern. Ther. Health Med..

[152] Abraham A., Hart A., Bozzorg A., Pothineni S., Wolf S.L., Schuh K., Caughlan M., Parker J., Blackwell A., Tharp Cianflona M. (2024). Comparison of Externally and Internally Guided Dance Movement to Address Mobility, Cognition, and Psychosocial Function in People with Parkinson’s Disease and Freezing of Gait: A Case Series. Front. Aging Neurosci..

[153] Jochum E., Egholm D., Oliveira A.S., Jacobsen S.L. (2024). The Effects of Folk-Dance in Schools on Physical and Mental Health for at-Risk Adolescents: A Pilot Intervention Study. Front. Sports Act. Living.

[154] Delabary M.D.S., Loch Sbeghen I., Teixeira da Silva E.C., Guzzo Júnior C.C.E., Nogueira Haas A. (2024). Brazilian Dance Self-Perceived Impacts on Quality of Life of People with Parkinson’s. Front. Psychol..

[155] Elpidoforou M., Bakalidou D., Drakopoulou M., Kavga A., Chrysovitsanou C., Stefanis L. (2022). Effects of a Structured Dance Program in Parkinson’s Disease. A Greek Pilot Study. Complement. Ther. Clin. Pract..

[156] Mehta A., Dugani P., Mahale R., Nandakumar, Haskar Dhanyamraju K., Pradeep R., Javali M., Acharya P., Srinivasa R. (2024). Garba Dance Is Effective in Parkinson’s Disease Patients: A Pilot Study. Parkinson’s Dis..

[157] Tillmann A.C., Andrade A., Swarowsky A., Guimarães A.C.D.A. (2017). Brazilian Samba Protocol for Individuals with Parkinson’s Disease: A Clinical Non-Randomized Study. JMIR Res. Protoc..

[158] Bocchi Martins J.B., Amaral da Rocha A.R., Fausto D.Y., da Silva E., da Silva G., Pinheiro G.P., de Azevedo Guimarães A.C. (2025). Comparing Urban Dance and Functional Fitness for Postmenopausal Women: A Randomized Clinical Trial Protocol. Eur. J. Obstet. Gynecol. Reprod. Biol..

[159] Koh Y., Noh G. (2020). Tango Therapy for Parkinson’s Disease: Effects of Rush Elemental Tango Therapy. Clin. Case Rep..

[160] Rodríguez-Quiroga S.A., Rey R.D., Arakaki T., Garretto N.S. (2014). Dramatic Improvement of Parkinsonism While Dancing Tango. Mov. Disord. Clin. Pract..

[161] Batson G., Hugenschmidt C.E., Soriano C.T. (2016). Verbal Auditory Cueing of Improvisational Dance: A Proposed Method for Training Agency in Parkinson’s Disease. Front. Neurol..

[162] Carapellotti A.M., Rodger M., Doumas M. (2025). Evaluating the Short-Term Effects of Dance on Motor and Non-Motor Outcomes in People Living with Parkinson’s: A Crossover Study. PLoS ONE.

[163] Hackney M.E., Earhart G.M. (2009). Health-Related Quality of Life and Alternative Forms of Exercise in Parkinson Disease. Parkinsonism Relat. Disord..

[164] Hu B., Wang X., Lu S., Ying X. (2025). A Study of Bidirectional Control of Parkinson’s Beta Oscillations by Basal Ganglia. Chaos Solitons Fractals.

[165] Westheimer O. (2008). Why Dance for Parkinson’s Disease. Top. Geriatr. Rehabil..

[166] Moratelli J., Alexandre K.H., Boing L., Swarowsky A., Corrêa C.L., Guimarães A.C.d.A. (2021). Binary Dance Rhythm or Quaternary Dance Rhythm Which Has the Greatest Effect on Non-Motor Symptoms of Individuals with Parkinson’s Disease?. Complement. Ther. Clin. Pract..

[167] Brunet J., Saunders S., Gifford W., Thomas R., Hamilton R. (2018). An Exploratory Qualitative Study of the Meaning and Value of a Running/Walking Program for Women after a Diagnosis of Breast Cancer. Disabil. Rehabil..

[168] Flannery M. (2017). Self-Determination Theory: Intrinsic Motivation and Behavioral Change. Oncol. Nurs. Forum.

[169] McMahon J., Chazot P. (2020). Dance and Parkinson’s: Biological Perspective and Rationale. Lifestyle Med..

[170] Saluja A., Goyal V., Dhamija R.K. (2023). Multi-Modal Rehabilitation Therapy in Parkinson’s Disease and Related Disorders. Ann. Indian Acad. Neurol..

[171] Bevilacqua R., Maranesi E., Benadduci M., Cortellessa G., Umbrico A., Fracasso F., Melone G., Margaritini A., La Forgia A., Di Bitonto P. (2025). Exploring Dance as a Therapeutic Approach for Parkinson Disease Through the Social Robotics for Active and Healthy Ageing (SI-Robotics): Results from a Technical Feasibility Study. JMIR Aging.

[172] Bevilacqua R., Benadduci M., Bonfigli A.R., Riccardi G.R., Melone G., La Forgia A., Macchiarulo N., Rossetti L., Marzorati M., Rizzo G. (2021). Dancing With Parkinson’s Disease: The SI-ROBOTICS Study Protocol. Front. Public Health.

[173] Goka K.A., Agormedah E.K., Achina S., Segbenya M. (2024). How Dimensions of Participatory Decision Making Influence Employee Performance in the Health Sector: A Developing Economy Perspective. J. Chin. Hum. Resour. Manag..

[174] Giménez-Llort L., Castillo-Mariqueo L. (2020). PasoDoble, a Proposed Dance/Music for People with Parkinson’s Disease and Their Caregivers. Front. Neurol..

[175] Croft S., Fraser S. (2021). A Scoping Review of Barriers and Facilitators Affecting the Lives of People with Disabilities During COVID-19. Front. Rehabil. Sci..

[176] Carretti G., Tognaccini A., Baldini G., Manetti M., Marini M. (2025). Kinesiological Insights into Exercise Prescription within Oncological Prehabilitation: Current Knowledge and Future Perspectives. Front. Sports Act. Living.

[177] Leonardi F. (2018). The Definition of Health: Towards New Perspectives. Int. J. Health Serv..

